# Towards an Enhanced Understanding of Plant–Microbiome Interactions to Improve Phytoremediation: Engineering the Metaorganism

**DOI:** 10.3389/fmicb.2016.00341

**Published:** 2016-03-16

**Authors:** Sofie Thijs, Wouter Sillen, Francois Rineau, Nele Weyens, Jaco Vangronsveld

**Affiliations:** Department of Biology, Centre for Environmental Sciences, Hasselt UniversityDiepenbeek, Belgium

**Keywords:** phytoremediation, metaorganism, contaminant biodegradation, plant growth promotion

## Abstract

Phytoremediation is a promising technology to clean-up contaminated soils based on the synergistic actions of plants and microorganisms. However, to become a widely accepted, and predictable remediation alternative, a deeper understanding of the plant–microbe interactions is needed. A number of studies link the success of phytoremediation to the plant-associated microbiome functioning, though whether the microbiome can exist in alternative, functional states for soil remediation, is incompletely understood. Moreover, current approaches that target the plant host, and environment separately to improve phytoremediation, potentially overlook microbial functions and properties that are part of the multiscale complexity of the plant-environment wherein biodegradation takes place. In contrast, *in situ* studies of phytoremediation research at the metaorganism level (host and microbiome together) are lacking. Here, we discuss a competition-driven model, based on recent evidence from the metagenomics level, and hypotheses generated by microbial community ecology, to explain the establishment of a catabolic rhizosphere microbiome in a contaminated soil. There is evidence to ground that if the host provides the right level and mix of resources (exudates) over which the microbes can compete, then a competitive catabolic and plant-growth promoting (PGP) microbiome can be selected for as long as it provides a competitive superiority in the niche. The competition-driven model indicates four strategies to interfere with the microbiome. Specifically, the rhizosphere microbiome community can be shifted using treatments that alter the host, resources, environment, and that take advantage of prioritization in inoculation. Our model and suggestions, considering the metaorganism in its natural context, would allow to gain further knowledge on the plant–microbial functions, and facilitate translation to more effective, and predictable phytotechnologies.

## Current Challenges in Exploiting Plants and Microorganisms for Phytoremediation

Phytoremediation is an environmentally sustainable, solar-powered, and cost-effective soil remediation technology which relies on the ability of plants to intercept, take-up, accumulate, sequestrate, stabilize or translocate contaminants (Pilon-Smits, 2005). Additional benefits of phytoremediation include the conversion of biomass for bioenergy, sustaining of biological biodiversity, soil stabilization, and numerous other ecosystem services. Contaminants that are taken-up by the plant, can be detoxified by plant secondary metabolism or via stimulation of plant-associated microorganisms (Pilon-Smits, 2005; Weyens et al., 2009b). The efficiency of phytoremediation is strongly dependent on the selection of the plant species (Vangronsveld et al., 2009), but also on environmental factors like contaminant concentration, soil pH, nutrient status, oxidoreduction potential (Sessitsch et al., 2013), and in particular the soil-and plant-associated microorganisms.

Currently, phytoremediation does not reach the level of a highly efficient, predictable and fast clean-up technology. This is shown by often variable outcomes at the field scale, slow and incomplete degradation, and long clean-up time (Vangronsveld et al., 2009; Mench et al., 2010; Gomes, 2012; Stephenson and Black, 2014). Many studies have focused on improving soil conditions, contaminant bio-availability and accessibility (Robertson and Jjemba, 2005; Makris et al., 2010; De La Torre-Roche et al., 2012), plant growth conditions (Canellas and Olivares, 2014; Kidd et al., 2015), and the exploration and exploitation of soil and plant-associated microorganisms during phytoremediation (Barac et al., 2004; Weyens et al., 2010a; Abhilash et al., 2012; Becerra-Castro et al., 2013). However, the approaches focused for a long time on each organism individually rather than an integrated metaorganism approach in an ecological perspective. Optimizing the plant and its associated microorganisms, the metaorganism, has shown to be successful to improve plant growth in agriculture (Mendes et al., 2013; Rout and Southworth, 2013; Berg et al., 2014), and in disease resistance (Berendsen et al., 2012) but so far, an integrated metaorganism approach that allows uncovering the interactions between plant host and potentially thousands of degradative microbial taxa naturally colonizing the host, has not been largely performed.

In analogy to the human microbiome, the plant microbiome is essential for the plant, protecting the host against invaders, for the production of essential vitamins, improving nutrient solubility, as reviewed (Mendes et al., 2013). It is argued that the plant microbiome extends the functional potential of the host. In addition, the microbiome is able to regulate the expression of plant traits which lead to an improvement of the plant physiological state (Mendes et al., 2013). Recent evidence also emphasizes that phytoremediation success is strongly depend on the plant microbiome activities (Hassan et al., 2014). The impact has potentially been underestimated. To improve phytoremediation, an enhanced understanding of the plant and microbial interactions and responses to contaminants are essential. One particular aspect is to better understand how a host assembles a beneficial microbiome, and how it functions, under contaminant stress.

Molecular data and ecological models have already let to the understanding how insects assemble and maintain a beneficial cuticular microbiome (Scheuring and Yu, 2012). Also in gut microbiome research, microbiome establishment and functioning has been thoroughly scrutinized (Rooks et al., 2014), which has already resulted in translation to useful therapies, but the concepts have never been explored for a complex rhizosphere environment, let alone a contaminated soil. Though by understanding how the host interacts, shapes and maintains its microbiome proficient in plant-growth promoting (PGP) and contaminant degradation, we strongly believe that a more targeted stimulation of phytoremediation is possible. In this review we discuss for the first time a competition-driven model to explain the assembly of a beneficial microbiome under contaminant stress. This is based on direct and indirect evidence from recent phytoremediation studies, integrated from a whole new perspective. The second part of the review addresses the questions whether the selected metaorganism is optimal for phytoremediation, and whether the microbiome is highly efficient for PGP and contaminant degradation? New challenges that are triggered from the model are discussed and we suggest improvements in the current approaches. Although we focus here on optimizing the metaorganism for phytoremediation, many principles of this competition-driven model, may apply to optimising plant–microbial interactions during crop production, bioenergy, and landscape management.

## A Competition Driven Model to Explain the Establishment of a Beneficial Microbiome in a Contaminated Rhizosphere

Explaining how microbiomes are established and maintained is a hot research topic in many disciplines, ranging from plant-pathogen research (Berendsen et al., 2012), gut microbiome research (Muegge et al., 2011) and community ecology (Gotzenberger et al., 2012), but also potentially holds the key to improving phytoremediation. Hosts in general receive a lot of benefits from their associated microorganisms, such as protection against invaders, enhancing nutrition and growth, improving resilience in stress conditions, as has been extensively reviewed (Mendes et al., 2013). In a study which used *Arabidopsis halleri* plants grown on a natural soil and a gamma irradiated soil, it was shown that when being grown on a natural soil the plants accumulated higher levels of cadmium and zinc in the tissues, indicating that microbiota are essential players during phytoextraction (Muehe et al., 2015). Moreover, it is increasingly accepted that hosts assemble non-random sets of microbial symbionts with a higher proportion of beneficial microbes than expected by chance. Also in the case of a contaminated soil, it appears that the plant host can select microbes with degradative genes out of a huge pool of candidates in the bulk soil (Siciliano et al., 2001; Sipila et al., 2008), but a detailed understanding how the host does this lacks.

To better understand the establishment of the microbiome in the plant rhizosphere/endosphere, this habitat is often compared to the gut microbiomes. Gut microbiomes are shaped by host species genotype and priority effects (transmission via parents; Ochman et al., 2010), diet (Muegge et al., 2011), and transplantation and disturbance (antibiotics; Rooks et al., 2014). In addition, recently Scheuring and Yu (2012) identified the conditions under which insects (attine ants) could successfully screen in a beneficial microbiome that was dominated by antibiotic producing and resistant bacteria. For this they distinguished three steps: first, the new host starts with a higher proportion of beneficial antibiotic producing microbiota, second, the ant host provides resources, which third fuel intense competition via antibiotic production and results in competitive dominance by antibiotic producing bacteria (Scheuring and Yu, 2012). Analogous, if we can identify the conditions under which the plant host in a contaminated soil can select degradative traits, than this can results in more targeted interfering.

To model the establishment of a beneficial microbiome in a plant host is much more complex than the ant and human gut microbiome models suggested so far. The plant host species itself (genotype) has a large influence on the microbiome composition, and evolves in ways (growth stage, disease state, herbivory etc.) that changes the microbiome. In addition to plant state and productivity, the presence of neighboring plants and in general species richness are thought to strongly influence the abundance of microorganisms belowground (De Deyn et al., 2011). Further, the abiotic environment has a large impact on the microbiome composition. Soil is much more complex biologically, in terms of species diversity, and external abiotic factors which adds numerous variables and uncertainty to the model. Previous attempts to model biodegradation have shown it is very hard to simplify the biodegradation space in units that define contaminant degradation (see **Box 1**). Though an ecological model which combines a metaorganism approach and reductionist views seems to be most suited (**Box 1**).

**BOX 1 | Complexity of contaminant degradation.** A recent pyrosequencing study analyzing bacterial community diversity in the permafrost soil along the China–Russia Crude Oil Pipeline, found then thousands of operational taxonomic units (OTUs) with 84,834 reads, numbers which by far outdid the expectations for such an extreme habitat (Yang et al., 2012). Similarly, Bell et al. (2013b) revealed a high diversity of hydrocarbonoclastic bacteria in the rhizosphere of *Salix* growing on hydrocarbon contaminated soil in Canada (Bell et al., 2013b). In line with this, recent genome insights revealed that aliphatic and aromatic catabolic genes are abundant in the root endophytic *Burkholderia* sp., even if they were not exposed previously to diesel fuel in their environment (Mitter et al., 2013). These studies suggest that catabolic genes are likely ubiquitous in the environment, though it remains to be shown whether they are active, and most proficient for soil remediation.

de Lorenzo (2008), showed that an abstraction of the biodegradation process with purely genetic and enzymatic reactions is not optimal for predicting biodegradation rates. That is because the degradation of any given compound, whether or not expressed on plasmids, is highly influenced by many upstream (bioavailability) and downstream factors (toxicity of intermediates, stress, nutrients). An illustrative example is the inability of strain *Burkholderia xenovorans* LB400 to completely degrade polychlorinated biphenyls (PCBs) despite having in its genome all genes which are necessary to do this (Pieper and Seeger, 2008). To further illustrate the complexity of biodegradation, contaminant degradation is usually not limited to specialist taxa but generalists are involved. In fact, co-metabolism of pollutants and interspecies metabolism is the rule rather than exception. Finally, multiple enzymes within a cell, and degradation pathways compete for the substrates, and thus biodegradation in a community is the result of the *pan-metabolome* (de Lorenzo, 2008).

To deal with this complexity, it is increasingly accepted that phytoremediation needs to be approached from a systems biology, and community ecology perspective. Caro-Quintero and Konstantinidis (2012), provided evidence from the metagenome level that a population and community approach potentially hold true for microbial populations, acting in distinct entities (Caro-Quintero and Konstantinidis, 2012). In addition, Sørensen et al. (2009) amongst others, pursue the understanding of the behavior of single cells in a large community to better understand functions at the population level (Sørensen et al., 2009; Remus-Emsermann et al., 2012). Only if we have scrutinized the single cell behaviour in complex soil microbial communities, combined with insights from the metaorganism functioning (pairing plant and microbe omics), than we can potentially identify and predict cross-species and cross-kingdom functions, that can be targeted to increase soil remediation. In the best possible scenario, we have a good catalog of the genome sequences and transcriptomes of all (or most important) players, which will allow to predict their metabolic activities (Larsen et al., 2011). Many studies have already obtained relevant insights at the metaorganism level, thanks to the ongoing developments in the high-throughput sequencing technologies. However, to further our understanding, integrating large sequencing data in models requires massive computational power, which is not always available. Nevertheless, improvements to current research strategies, considering *in situ* conditions (see **Table 1**), would allow to gain further insight in the functioning and significance of the microbiome for phytoremediation, potentially leading to the design of improved treatments that specifically promote highly efficient biodegradative communities.

Here, we propose that an adapted model of Scheuring and Yu (2012) can explain some of the driving factors under which conditions plants directly or indirectly promote the abundance of PGP and degradative bacteria in a contaminated soil. To demonstrate the model concept, we will focus on the plant rhizosphere, though potentially the same interactions occur in the phyllosphere, and plant-endosphere habitats. More in particular our attention will be focused on the bacteria and mycorrhizal fungi in the rhizosphere, which are by far the best studied microorganisms in relation to phytoremediation. Plant-growth promoting rhizobacteria (PGPR) and mycorrhizal fungi live in a mutualistic symbiosis with the host. The plant provides root exudates and creates habitats for the microorganisms, and, in return, the PGP degradative bacteria and mycorrhiza promote plant growth and detoxify the contaminants.

### The Model

We propose a competition-driven model, based on the theory described in Grime (1977) and the recent findings of Yergeau et al. (2014), to explain the establishment and maintenance of a beneficial microbiome in the plant rhizosphere on contaminated soil, and suggest that (1) if plants provide abundant resources in the rhizosphere this will favor interactions that confer competitive advantage to degradative (and tolerant) microorganisms depending on the contaminant concentration level, and that (2) the establishment of such a beneficial degradative microbiome is more efficient if there is some kind of vertical transmission (seed transfer of degradative traits) or high abundance of degradation genes in the microbial pool at early stages of colonization of the soil. Thus, a higher immigration rate of degradative (and tolerant) microbiota from the environment, e.g., in response to specific root exudates secreted by the host, combined with competitive interactions which favor the screening in of these phenotypes, give shape to the rhizosphere microbiome in a contaminated soil. Evidence to support the competition-driven model is provided below.

### Contamination Influences the Rhizosphere and Root Microbial Community

Siciliano et al. (2001) investigated the endosphere and rhizosphere microbiomes of different grass species growing on a petroleum and nitroaromatic contaminated soil, and they revealed that contaminant concentration was a major factor that determined rhizosphere and root-endosphere microbiome structure and function. Moreover, there was a plant-specific and selective effect on the prevalence of specific catabolic genes, e.g., tall fescue rhizosphere community was characterized by an enrichment of catabolic genotypes such as alkane monooxygenase (*alkB*), naphthalene dioxygenase (*ndoB*), and nitrotoluene monooxygenases (*ntnM*; Siciliano et al., 2001), but there was a decrease in the prevalence of catabolic genes in the rhizosphere of rose clover (Siciliano et al., 2003). This indicates that plants can exert control over microbial degradative traits in the rhizosphere, and hence phytoremediation activity. The presence of plants also influenced the prevalence of degradation genes outside the rhizosphere, in the surrounding bulk soil (Siciliano et al., 2003). Another study showed that the presence of diesel in soil significantly explained microbial community composition and diversity, even overruling soil matrix type effects (Sutton et al., 2013). A number of other recent studies confirm the initial findings, that the contaminated rhizosphere microbiomes often contain a higher abundance of catabolic genes as determined by qPCR (Sipila et al., 2008), which are also actively expressed as shown by a metatranscriptomics analyses (Yergeau et al., 2014). These observations raise the question how the plant host ‘selects’ degradative microbiota or rather traits, given the enormous pool and variety of microorganisms in soil.

One explanation, which is still heavily debated though, is that the beneficial plant microbiota for a part naturally coevolved with host (e.g., via vertical transfer of degradative traits via the seeds; Mastretta et al., 2009; Hodgson et al., 2014), or coevolution of mycorrhiza with the plant species with selective recruitment from the environment (Hoeksema, 2010; Davison et al., 2011). A recent study has shown that vertical transmission of fungal endophytes from mother plant to offspring, via seeds, is common in forb species (e.g., *Papaver rhoeas, Plantago lanceolata* etc.), suggesting that this may be a widespread phenomenon (Hodgson et al., 2014). The transferred endophytes can influence seedling germination rate, provide protection against herbivores and pathogens, and stress resilience depending on the endophytes that were present in the mother plant. Another study showed that plants grown on cadmium soil contained a high prevalence of seed-endophytes which produced IAA, ACC-deaminase and displayed Cd tolerance, *in vitro* (Truyens et al., 2015), and these traits were transferred from generation to generation (Truyens et al., 2013). The other observation, whether there is ongoing co-evolution of mutualistic fungi and the host, is currently heavily debated (Hoeksema, 2010). De Deyn et al. (2011), showed that the abundance of arbuscular mycorrhiza (AMF) is strongly influenced by plant species diversity, richness and identity, which indicates that coevolution to some extent may be ongoing. In fact, ectomycorrhizal fungi (EMF) and AMF are crucially important in protecting plants from toxic metals (Colpaert et al., 2011), and organic pollutants (Bonfante and Anca, 2009), protect physically the roots (Barea et al., 2002), and the radicals or catabolic enzymes produced by the fungi can be involved in the detoxification of contaminants (Harms et al., 2011).

However, if vertical transmission and coevolution would be the only process, it would make it very difficult to explain why similar plant cultivars in different soils contain different microbial communities, with different phytoremediation activities. The explanation is that the rhizosphere microbiome is recruited for a large part from the surrounding soil environment (Haichar et al., 2008; Berg and Smalla, 2009). In fact, plants constantly recruit a diverse set of microbiota from the soil environment, which may display degradative potential with different modes of action and PGP potential to variable extents (Croes et al., 2013; Bell et al., 2014; Yergeau et al., 2014; Truyens et al., 2015). Hence, it is not unexpected that the host evolves a beneficial degradative microbiome differently at different places (Lebeis, 2014). What is the major mechanism underlying the selective recruitment?

### Root Exudates Change under Contaminant Stress: Plants Call for Support?

It is well known that root exudates play a large role in shaping the rhizosphere microbiome (Berg and Smalla, 2009). Microbiota compete for the abundant resources (Raaijmakers et al., 2008), and thereby specific exudates can selectively attract or repel certain species (Neal et al., 2012). Berendsen et al. (2012) have suggested that plant beneficial traits may be selected for because plants secrete specific root exudate compounds in response to pathogen attack, selectively recruiting protective microorganisms. Adoption of this idea to a contaminated soil, hypothesizes that plants change root exudates in response to contaminants, to favor degradative traits (‘call for support’). In fact, a number of studies have shown that plants alter the quality and quantity of rhizodeposits in response to contaminants (Zhou et al., 2011). One notable study has demonstrated, using a split-root model, that certain plant species respond in a systemic manner to PAH-contamination (indirect effect) and that the root-exudate induced changes in microbial community composition was correlated with abundance changes of particular genera that are suggested to play an important role in rhizoremediation (Kawasaki et al., 2011). However, some researchers argue that root exudates are not actively but passively released from plants, and that the host does not actively recruit microbial species or traits (Hartmann et al., 2008). The competing explanation for the observed mutualism, is that if the hosts creates a selective and demanding environment such that the habitat turns out to be attractive to mutualists and symbionts, and unattractive to pathogens or parasites, then a selective recruitment of mutualists occurs, independent whether the host knows the quality and quantity of the individual symbionts (Archetti et al., 2011). Hence, if the conditions are set right by the host, the microbial symbiont evolves to accept the host or reject it (remain free living). Either of the opinions can be reconciled in which interactions between the partners could lead to a higher immigration of degradative microorganisms from the environment or specific secretions by the host or associated mycorrhiza (in secondary line) that could impart a competitive advantage to microbes that carry the appropriate detoxification genes. In this double selective environment, the resource availability can favor degradative PGP over pathogens.

### Competition as Driving Force

We add to the rhizosphere model, competition. It is well known that the rhizosphere is a highly competitive and selective environment because of the abundant resources provided by the host (Kowalchuk et al., 2002; Haichar et al., 2008; Berg and Smalla, 2009). Yergeau et al. (2014) suggested that a contaminated rhizosphere is more selective than a non-contaminated rhizosphere, based on metatranscriptomics data. They stated that under the double selective pressure, only microorganisms that can use the specialized carbon sources provided by the host, and can cope with the presence of the contaminants, are highly competitive, and these will be significantly stimulated by the host (Yergeau et al., 2014). Evidence was based on the observation that the willow rhizosphere microbiome on hydrocarbon contaminated soil, showed a significant upregulation in genes coding for proteins involved in bacterial interference competition, biofilm formation, quorum sensing, and genes related to nutrient acquisition compared to a non-polluted soil (Yergeau et al., 2014).

**FIGURE 1:**
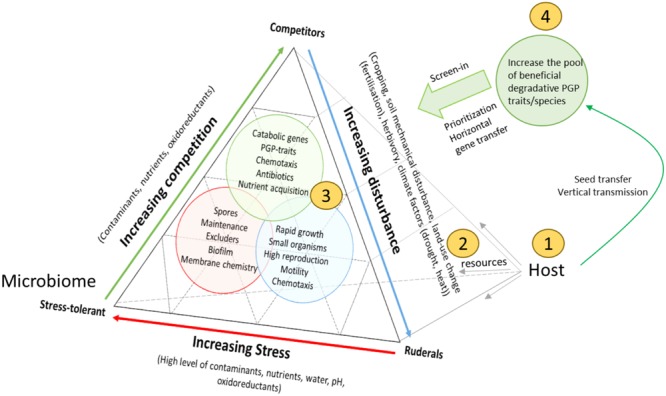
**Schematic representation of the different interactions taking place between the host and its rhizosphere microbiome, and possible ways to interfere with rhizosphere microbiome functioning for phytoremediation.** The figure is adapted from Krause et al. (2014) who extended the competition-tolerant-ruderal life strategy of plants to microbial communities in soil. We add the plant-host to it, and use this framework for assigning life-strategies to rhizosphere microorganisms: (S) stress-tolerant, (C) competitors, (R) ruderals, and many intermediate microbial life strategies. It is assumed that the host fuels the interactions by providing abundant resources. S, C, or R can represent single species or functional groups of microorganisms (consortia), and the terms are used to distinguish S (contaminant-tolerant species), competitors (degradative competitors, exploitation competitors, or interference competitors), and ruderals (rapid growing species). Although the plant-provided resources (plant exudates) fuel higher growth rates of all microorganisms, some of the resources can be partitioned more to beneficial, degradative microorganisms that outcompete others (e.g., by capitalizing unique carbon sources, producing antibiotics, etc.). As such, it can be understood that degradative traits may be selected for by the host, if it confers a competitive superiority in the rhizosphere niche, meaning that biodegradation is ideally coupled to growth (central metabolism) and detoxification. If contaminant concentrations are low, plants may preferable recruit PGP, while commensals and pathogens capitalize on the resources. Which equilibrium is reached depends on which microbial type has the higher abundance and functional activities. Moreover, the community can also display priority effects: it is much more difficult for pathogens/opportunists to invade a community already dominated by primary colonizers (e.g., catabolic strains). Compared to an idealized model, the effectiveness of contaminant degradation may be limited by the presence of opportunists that do not contribute to biodegradation but consume the same resources, or detrimental strains that inhibit the activity of catabolic strains. Disturbing the communities in their niche, by selecting the host species (1), manipulation of their nutrients (root-exudates) (2) and corresponding niches (by disturbance) (3), or the introduction or removal of key members (4), may significantly alter the balance and strength of interactions between them. These interventions can cause changes in the way that microorganisms can influence each other, their niche, and the host, with consequences for phytoremediation activity.

To ground the competition model further, we refer to the ideas of Grime (1977), who suggested three primary strategies of viable plant habitats. Grime (1977) defined two external factors, ‘stress’ (conditions that restrict plant growth, e.g., shortage of water, and nutrients), and disturbance (partial or total destruction of plant biomass, e.g., herbivory, pathogen attack, tillage, weather conditions), and permutations of both factors resulted in the delineation of three viable plant life-strategies: (1) low stress with low disturbance, favors competitive plants, (2) high stress with low disturbance, selects for stress-tolerant plants, and (3) low stress with high disturbance, provides advantage to ruderal plants (Grime, 1977). A competition-based reflection of Grime’s idea to rhizosphere microbial communities would hypothesize that a degradative microbiome can only evolve when contaminant concentrations are moderate to low, and degradation confers a competitive advantage in the rhizosphere niche. High pollution levels with low disturbance would select contaminant-tolerant microbial phenotypes (**Figure 1**). Though, if contaminant concentrations are diffuse but the bulk soil was/is heavily disturbed (e.g., by excavation, tillage, chemical treatment, fertilization, etc.) this would favor ruderal microorganisms which respond rapidly to free niches and the presence of abundant, easy carbon sources (e.g., r-strategists), but may not contribute to biodegradation (opportunists). Consistent with this scenario, it has been shown that rapid-growing *Pseudomonas* species and Alphaproteobacteria (r-strategists) flourish in disturbed contaminated environments and capitalize on the resources (Yergeau et al., 2014). However, microorganisms likely have many more life-strategies than r/K and probably switch between life-strategies (Fierer et al., 2012). Previously, a similar approach has been used to better understand microbial biodiversity in ecosystem functioning (Krause et al., 2014). Moreover, recent evidence from the metagenomics level, supports that microbial communities are organized in genetically and ecologically discernible populations, which possess the attributes expected for species (Caro-Quintero and Konstantinidis, 2012). In this sense, a reflection of microbial traits on the competitor-ruderal-stress tolerant life-strategy framework from a community ecology approach of Grime (1977), may explain some of the interactions that take place in a contaminated rhizosphere. It is to note that the rhizosphere microbiome will not always be in one of the extremes, but represents a compromise between conflicting selection pressures resulting from particular combinations of competition, stress, and disturbance (**Figure 1**).

## Towards a Plant-Metaorganism Approach in Phytoremediation: Practical Approaches

We have proposed a competition-driven model for the rhizosphere microbiome to understand and identify some of the factors that drive the assembly of a beneficial microbiome under which PGP and degradative microbiota have a competitive advantage in a highly contaminated soil. Although the competition-based model may appear oversimplified, and incorporating all partners (next to bacteria and fungi, also archaea, arthropoda, protista, and other macro- and mesofauna etc) will better represent phytoremediation activity, the model shows the inherent interactive character of plant–microbial interactions for contaminant degradation, and provides a basis for the development of improved and more effective phytoremediation strategies. In fact, a naturally evolved rhizosphere microbiome may not be the most optimal for phytoremediation activity. For degraders to abundantly colonize the niche, there should be a high dominance at the start of the competition (niche colonization), so that enough degraders are present to ward-off opportunists that do not contribute to degradation. Moreover, loss of sensitive taxa can severely alter the way the plant host interacts with the other microbiome members (Bell et al., 2014). Furthermore, the selected host species can in some cases be incompatible for inoculation with certain types of bacteria or fungi, because they are not naturally selected for by the host. Furthermore, despite the presence of degradative genes in the natural soil microbial communities, the persistence of recalcitrant organic pollutants in soil, indicates that natural attenuation is insufficient, and hence requires interfering by men.

With the model in mind, we can see that many new opportunities have arisen to optimize the metaorganism in phytoremediation, rather than the plant and microbe part separately (**Figure 1, Table 1**). Multiple strategies can be considered to prevent opportunists and pathogens from winning over degradative and PGP microbes. Four strategies are proposed which are likely to be important in redirecting the microbiome: (1) Selecting plants not only for high biomass/rapid growth rate, and tolerance, but also for their global interaction with the microbiome, (2) changing the root exudates (microbial diet), (3) taking into account vertical transmission and promoting a higher ‘immigration’ rate (feeding the supply lines, priority effects), and (4) altering the competitive interactions that evolve between host–microbe, microbe–microbe, and microbe–environment (applying disturbance). Example research strategies and practical approaches how to take into account this metaorganism concept is given in **Table 1**, and is further discussed in the following paragraphs.

**Table 1:** Possible ways to improve phytoremediation research using a metaorganism approach.

	Metaorganism approach	Example research strategy
Selecting the right plant host	In function of the microbiome	A pyrosequencing study identified that native willow cultivars were associated with a different microbial community than non-native cultivars, across a hydrocarbon contaminated soil (Bell et al., 2014). Fungi were more sensitive to hydrocarbon contamination then bacteria, and reacted different to willow introduction, suggesting that plant species selection (and evolutionary history) are not to underestimate with regard to their effect on microbiome establishment and influence on phytoremediation activity.
	Subsequent microbial inoculation practices	Fungi grown in their soil of origin with native plant species have been shown to be more mutualistic (more arbuscules), which can enhance the introduction of cooperative strains at a later stage (Johnson et al., 2010)
	Breeding, and transgenesis of plants for high biomass/rapid growth, high tolerance, uptake and detoxification potential	Transgenic tobacco cultivars over-expressing a bacterial nitroreductase resulted in improved TNT detoxification, and additionally increased the functional diversity of the rhizosphere microbial community (Travis et al., 2007), a double positive effect which warrants further investigation.
Interfering with root-exudates (diet)	Rhizoengineering	There are promising outlooks to change the quality and quantity of root exudates in the rhizosphere to optimize plant growth (Zhang et al., 2015) and biodegradation (Narasimhan et al., 2003). Transgenic *Arabidopsis* plants that exuded the xenotopic compound octopine, significantly increased the ratios of octopine degraders (Mondy et al., 2014).
	Selecting plant traits for their global interaction with the rhizosphere microbiome	Maize seedlings were shown to exude a high concentration of the compound DIMBOA which exerts antimicrobial activities in the rhizosphere (Neal et al., 2012). In addition, DIMBOA also attracts a catabolic (Neal et al., 2012), but also recruiting a catabolic, plant-beneficial rhizobacterium *Pseudomonas putida* KT2440. Can we identify more plant traits that globally interact with the rhizosphere microbiome?
Modify the driving forces	Elucidating the main factors that influence the plant–microbiome interactions	Antibiotic administration altered the community structure of gastrointestinal microbiota (Robinson and Young, 2010), similarly antibiotic addition to soil changed the soil microbiome leading to increased hydrocarbon degradation rates (Bell et al., 2013a). It is assumed that reduced interspecies competition enhances the catabolic activity of degradative strains.
	Studying interactions at the single microbial cell level *in situ*, and extrapolate/confirm findings at the population and community level (bottom-up).	Remus-Emsermann et al. (2012), used gfp-tagged individual *Erwinia herbicola* cells as bioreporter, to better understand bacterial colonization of the leaf surface of *Phaseolus vulgaris* plants. They suggested that the ‘carrying capacity,’ can be understood as the sum of local carrying capacities (Remus-Emsermann et al., 2012) whereby leaves contain a few sites where individual cells can produce high numbers of offspring, and the remainder of the leaf offers sites with low and medium reproductive success. Using such a bottom-up approach in phytoremediation, can help to better understand bacterial colonization of the rhizosphere at the population and community level.
Feeding the supply lines	Isolation of previously difficult to culture degradative strains	Continued efforts in culture-based techniques including the use of improved culture media and intelligent devices such as the i-Chip, have enabled the cultivation of a broader collection of previously difficult to cultivate microorganisms (Nichols et al., 2010; Stewart, 2012).
	Selection of strains/consortia to inoculate	The identification of the core rhizosphere microbiome and core root microbiome (Lundberg et al., 2012; Yeoh et al., 2015), i.e., strains that sufficiently depend on host genotype, but remain consistent across different soil types and developmental stages, allows selecting strains for their intimate interaction with the host, which should facilitate their introduction/enrichment in the native microbial community.
	Time point of inoculation matters, prioritization	Preemptive colonization of plant leaves with beneficial bacteria was found to reduce, but not completely exclude, the ability of secondary colonizers to reproduce and proliferate (Remus-Emsermann et al., 2013). These findings have direct relevance to phytoremediation, based on preemptive exclusion of opportunists and pathogens when there is a high level of catabolic strains at the start.
	Exploiting horizontal gene transfer	Introduction of the endophyte *Burkholderia cepacia* VM1468, equipped with the pTOM-Bu61 plasmid coding for toluene degradation, in the rhizosphere of yellow lupine has shown to dramatically reduce toluene evapotranspiration. Interestingly, the catabolic plasmid was found to transfer from the inoculated strain to different members of the endogenous plant endophytic community (Taghavi et al., 2005). As such harnessing horizontal gene transfer is a simple and inexpensive way to enrich catabolic traits.

## Strategy 1: Selecting Plants in Function of the Microbiome

Initial accounts in phytoremediation focused on plants with high biomass and fast-growth, tolerance and/or high accumulation capacities (hyperaccumulators). However, focusing on these traits has moved away the attention to selecting plants as a tool to change and influence the associated microbiome, which has a substantial impact on phytoremediation outcome (Hassan et al., 2013). In fact, it has been shown that transgenic crops (e.g., genetically modified Bt-maize selected for higher disease resistance) have a less diverse (simplified) rhizosphere microbiome showing a lower level of mycorrhization and both the total and active heterotrophic bacteria (Castaldini et al., 2005). The potential adverse consequences of plant domestication (Pérez-Jaramillo et al., 2015) on non-target rhizosphere microbial communities and the long term resilience, warrants further investigation. Selecting the plant species (genotype or cultivar) determines for a large part microbial community structure and function (Berg and Smalla, 2009), and thus plant selection is an important tool to modify plant-associated microbial communities. Therefore, alternative plant-based selection strategies that integrate microbiome functions by promoting the assembly of specific plant-associated microbial communities is a novel opportunity to improve phytoremediation and biomass yield.

Practically, one can evaluate the plant-effect on the microbiome from an explorative approach, but also by proposing interventions aimed to benefit the microbiomes and phytoremediation outcome (manipulative; Cravatt and Kodadek, 2015). For instance, Hassan et al. (2014) recently used pyrosequencing of amplified 16S rRNA from the rhizosphere of 11 willow cultivars across hydrocarbon contaminated soil, to show that rhizospheric AMF community structure varies between the willows and that different symbionts are involved in plant adaptation to hydrocarbon contamination (Hassan et al., 2014). Moreover, contaminant concentration had a main effect on AMF community structure with different AMF families dominating at each contaminant level. In another study, it was shown that not only the introduction of willow species in hydrocarbon contaminated soil increased the diversity of fungal communities, but also that the community composition diverged when *Salix* genotypes were less closely related (Bell et al., 2014).

Recently, the introduction of non-native plant species in soil has been shown to form less beneficial associations with mycorrhizal fungi than native plant species, and this may reduce phytoremediation activity (Johnson et al., 2010). Indeed, also others have found that the health of locally adapted and foreign cultivars may be different depending on the strength of their associations with indigenous mycorrhiza (Philippot et al., 2013; Bell et al., 2014). These data suggest that the evolutionary history of plants should be considered when selecting plant varieties for phytoremediation and also the origin of cultivars. Pérez-Jaramillo et al. (2015) suggest a “back to the roots” framework that comprises the exploration of the microbiome of indigenous plants and their native habitats for the identification of plant and microbial traits with the ultimate goal to reinstate beneficial associations that may have been undermined during plant domestication (Pérez-Jaramillo et al., 2015).

Bell et al. (2014), pointed out that plants used in phytoremediation should also be selected so that the associated mycorrhizal partners have a supporting effect, or at least no antagonistic effects, on the inoculated bacteria (Bell et al., 2014). In this respect, better understanding of highly intimate plant-mycorrhizal fungi relationships (Hoeksema, 2010), could allow a better predictability of potential positive or negative interactions with other microorganisms such as bacteria. In addition, modifications of the established rhizospheric and endophytic microbial communities may enhance the introduction of subsequent strains.

## Strategy 2: Interfering with the Diet (Root Exudates)

During the past decade, there has been a considerable effort to characterize the chemicals that coordinate the establishment of the symbiotic interactions in the rhizosphere (Puschenreiter et al., 2005; Michalet et al., 2013), but despite this increased knowledge redirecting rhizosphere microbial communities with the current tools is still not trivial (Quiza et al., 2015). The nature and quantity of rhizodeposits is highly dynamic and varies depending on the plant species, the physiological stage of the plant, the presence or absence of plant neighbors, soil characteristics, soil contaminants, the soil microbial community context etc. (Chaparro et al., 2013). As a consequence of the complexity, only the major root exudate compounds have been identified. Though innovative tools like the micro-suction-cup technique (Puschenreiter et al., 2005; Oburger et al., 2013) allow *in situ* sampling of a broader spectrum of root exudates, which would allow gaining further knowledge of the potential function and significance of root educates in phytoremediation.

Because rhizodeposits play an important role in ‘selecting’ rhizosphere microbiota (Dennis et al., 2010), there has been a major interest in changing the quality and quantity of root-exudates via plant breeding and genetic modification to selectively stimulate specific microbial colonization, a technique called rhizoengineering (Van Aken et al., 2010; Quiza et al., 2015). Rhizoengineering was based on the early observations of *Agrobacterium tumefaciens*, which infects the host plant and induces a tumor (gall formation) that excreted opines, unusual carbon sources, that initially may have been used exclusively by the inducing crown gall bacterium (Dessaux et al., 1987). This work was followed by several rhizoengineering approaches based on the favorable partitioning of opines (Oger et al., 1997; Savka and Farrand, 1997; Narasimhan et al., 2003). Narasimhan et al. (2003) showed enhanced depletion of PCBs by the rhizobacterium *Pseudomonas* PML2, that utilized phenylpropanoids in plant exudates as growth substrate, in the rhizosphere of *Arabidopsis*. However, also other rhizobacteria that do not necessarily harbor the metabolic enzymes for the efficient catabolism of pollutants have been shown to utilize the specific plant secondary metabolites for growth. Hence, the sensitivity and specificity of these approaches should be improved. One possible solution is the identification of plant traits that interact with specific microbial community members (Smith et al., 1999; Remans et al., 2007; Neal et al., 2012). For instance, working with inbred lines of tomato plants, Smith et al. (1999), discovered the genetic basis for interactions with the beneficial rhizobacterium, *Bacillus cereus*, associated with plant growth and disease suppression. Similarly, Remans et al. (2007), identified two quantitative trait loci (QTLs) involved in auxin sensing in common bean, which could be used as a screening method for QTLs responsive to auxin producing bacteria. These studies hold promise for the use of genetic variation in plant species to enhance beneficial associations of plants with rhizosphere microbiota (Remans et al., 2007; Peiffer et al., 2013). To our knowledge there are no plant breeding strategies yet that have evaluated plant lines for their broad interaction with the rhizosphere microbiome. In this regard, it has been speculated to design a minimal rhizosphere microbiome (Raaijmakers, 2015), in analogy to the minimal genome (Juhas et al., 2011), with the final aim to select only these microorganisms (or traits) which are proficient for soil remediation and plant health.

## Strategy 3: Modifying the Driving Forces (Disturbance)

Although competition can favor the screening in of beneficial PGP and degradative microorganisms in the rhizosphere, and metagenomics data confirm that many of the microbial genes required in phytoremediation are already present in the environment (Sessitsch et al., 2012), in some cases this is not sufficient to attain high biodegradation activity. The occurrence and maintenance of contaminant degradation is the result of various competitive interactions including interference competition and resource exploitation, next to cooperative interactions like co-existence, mutualism, symbiosis, etc. that act on the partners, in space and time (Kowalchuk et al., 2002). The identification and understanding of such forces between the host and its microbiome, is important in order to optimize the metaorganism. Improving the understanding of the relationships between plant and its host poses novel challenges like (i) designing appropriate studies that aim to understand if relationships are direct (causal) or indirect and that may allow the creation of new conditions, and (ii) solving issues of assessing and statistical testing when analyzing relationships between the host and its microbiome (e.g., dynamics, feedbacks, uncertainties) in statistical models (**Figure 1**).

The final phytoremediation outcome is often assessed using chemical analyses, but this does not provide direct information on the contribution of plant–microbial processes or how management could be adapted to increase phytoremediation efficiency. To get a profound understanding of the consequences of environmental variables as drivers of changes in community composition and function, it is important to distinguish between direct and indirect relationships, as both can have trade-offs on contaminant degradation. In a direct relationship, changing one parameter (e.g., increasing soil fertility), would decrease or increase the second (e.g., increase crop yield), assuming the absence of other driving forces. However, host–microbe systems are statistically associated (negatively, positively, mixed) because their underlying drivers are statistically linked or correlated (indirect relationships). Both types of relationships are often referred to as ‘interactions’ independent of their correlative or causal nature. Conclusion about the nature of links between the host and its microbiome can be derived from studies using large, replicated datasets, and that use ‘manipulative’ approaches that enable to better understand and translate detailed knowledge of distinct biochemical processes into useful technologies. Experimental testing in the lab is then used to further explain the causal or correlative nature (Larsen et al., 2011). For example, to study the relationships between host and microbes, techniques such as the addition of fungicides/antimicrobial compounds (Bell et al., 2013a), nutrient disturbance (Bell et al., 2013b), and tillage (Treonis et al., 2010) have been used. Antibiotics addition (gentamicin and vancomycin) to hydrocarbon-contaminated soil was found to reduce bacterial and fungal abundance but to increase hydrocarbons degradation rate, confirming that bacterial-fungal competition in soil (Boer et al., 2005; Mille-Lindblom et al., 2006; Hibbing et al., 2010; Lecomte et al., 2011) is a strong influential force that also impacts biodegradation. Other studies that used cycloheximide, chloramphenicol (Myrold and Posavatz, 2007) and vancomycin (Robinson and Young, 2010) found similar effects on fungal and bacterial community structure, and associated function (Siciliano et al., 2009). Soil tillage and nutrient amendments promote homogenization, and this mostly favors generalist taxa that are adapted to the averaged conditions of the soil, in disadvantage of specialist taxa that only thrive in very specific micro-niches (Mangalassery et al., 2015).

Network models are frequently used to illustrate the nature of relationships between biological taxa and/or environmental variables (Ganter et al., 2013; Bolouri, 2014). Useful insights can come from natural, low complexity communities that may enhance the understanding of more complex systems. For example, in wastewater treatment plants it has been shown that biological interactions and taxonomic relatedness are dominant factors in explaining bacterial community assembly, while environmental variables such as sludge retention and inorganic nitrogen only partially explained the phylogenetic variances (Ju and Zhang, 2015). Negative co-excluding correlations were observed between less related species which probably indicated competitive interactions (Ju et al., 2014), and this has also been observed in soil ecosystems (Goberna et al., 2014). Other studies revealed a direct effect of nutrient or pollutant disturbance on the number of active catalytic microbiota in the rhizosphere that may lead to reductions in the specificity of plant–microbiota interactions (Stefanowicz et al., 2008; Bell et al., 2014; Sillen et al., 2015). Statistical models that link these processes are rapidly emerging (Vanwonterghem et al., 2014) illustrating this is becoming an intensively investigated research area.

Phytoremediation is also focused on predicting contaminant degradation and the consequences of future management options. Here, statistical models can be used to predict system shifts and fluctuations in phytoremediation activity as a consequence of environmental change (e.g., contaminant concentrations drop, plant growth stage, seasonal differences, etc.) and anthropogenic intervention. In this respect, different types of statistical, metabolic and ecological models are in use, but in general further development and refinement is necessary for obtaining reproducible results in their translation (Jimenez et al., 2014). For example, models based on niche preference and metabolic properties, can be used to predict microbial processes in nutrient removal from wastewater treatment plants, and allow to stimulate beneficial microorganisms and remove pathogenic and competing microbes (Vanwonterghem et al., 2014). Bell et al. (2013b), showed that the addition of nutrients to a hydrocarbon contaminated soil let to predictable shifts in microbial community structure, and associated degradation of petroleum hydrocarbons. In other studies, it was found that the presence of 2,4,6-trinitrotoluene (TNT) in soil promoted the relative abundance of *Pseudomonas* in a military soil in Flanders (Belgium; Thijs et al., personal communication) and this genus appeared to dominate as well in a TNT-contaminated soil in Spain (George et al., 2008), France (George et al., 2008), and UK (Travis et al., 2008a,b). If more studies become available, we can better understand why TNT-induced community shifts can evolve similarly under novel conditions, and this can in turn lead to novel treatment therapies. These examples demonstrate that the use of models is highly instructive to elucidate the main driving forces. It is to note that the models can never be better than the assumptions underpinning them. Furthermore, evaluation of uncertainties, which stem from uncertainty in the ability to capture relevant processes (e.g., plant-AMF ongoing co-evolution) as well as translating and scaling the information, need to be incorporated in current and future models.

## Strategy 4: Feeding the Supply Lines

In some cases, it is reasonable to assume a low abundance of degradative traits in the surrounding bulk soil. Nevertheless, a higher frequency of beneficial PGP and degraders can still be achieved, if the frequency of degradative traits or genera is increased (enriching, inoculation), resulting in a higher net immigration from the environment by competitive interactions (**Figure 1**). However, as there is a strong competition in soil for the introduced microbe to be accepted in the naturally occurring microbiome, strain inoculation is challenging. The ‘first-generation’ inoculants were not always targeted, reflected by variable outcomes in the field (Goldstein et al., 1985; Thompson et al., 2005; Violle et al., 2010). A deeper molecular-ecological understanding of the relationships between plants and microbiota is necessary to provide more targeted inoculation approaches, termed here ‘next generation inoculants.’

The first challenge is the ability to culture catabolic strains for inoculation. For this, detailed insights at the genetic level can provide useful information (Loper et al., 2012). For example, as reviewed by O’Brien et al. (2013), information from the genetic level can be used to predict the functional proteome and metabolism, and from this the formulation of the growth medium can be optimized to allow the cultivation of strains previously recalcitrant to cultivation (O’Brien et al., 2013). In addition, the approach of ‘taking the microorganisms back to the environment’ to grow them, e.g., using diffusion sandwich systems (Nichols et al., 2010; Stewart, 2012) or micro-Petri dish systems (Ingham et al., 2007), are promising tools which have proven to increase the recovery and diversity of growing isolates, even from the rare biosphere (Shade et al., 2012). In addition, since the genomes of representative PGPB such as *Pseudomonas, Burkholderia, Arthrobacter* (Wu et al., 2011; Mitter et al., 2013; Gkorezis et al., 2015) and AMF including *Rhizophagus irregularis* (Tisserant et al., 2013) have been sequenced, this has led to the identification of numerous genes and gene-clusters that determine plant-associated life-styles and which can be exploited in inoculant practices. Promising results have also been reported of studies that use mixtures of different PGP-strains with complementary actions, for example selected mixtures of trichloroethylene-degraders and trace element resistant PGB to tackle mixed pollutants (Weyens et al., 2015), PGPB and phosphate-solubilizing bacteria, PGPB and rhizobia, PGBR and endophytes (Rylott, 2014). In addition, partnering of mycorrhizal fungi with mycorrhizal helper bacteria (MHB) has shown promising results in agriculture (Frey-Klett et al., 2011), and certainly warrants further investigation in phytoremediation. Furthermore, combined biostimulants based on PGPB with plant extracts, humic acids, strigolactones, nod-metabolites, etc. has not been widely explored in phytoremediation compared to agriculture, where it demonstrated promising results on plant growth (Jayakumar et al., 2007). The hypothesis is that synergistic effects occur when formulations of living microbes and organic substances in specific combinations are applied to plants (Bakker et al., 2012; Mendes et al., 2013). Understanding the mechanisms of these complex interactions is not an easy task, but it opens a new avenue in the field of inoculants.

The second challenge is the time point and frequency of inoculation. Great efforts have already been put into getting more target-specific placement, timing and frequency of inoculation under different soil substrates (Afzal et al., 2011, 2013), and determining the optimal bio-inoculant concentration (Weyens et al., 2012), but the variable outcomes indicate that our understanding is yet incomplete. A particular important aspect to improve is taking into account prioritization, which means introducing beneficial microorganisms from the start to achieve niche saturation and competitive exclusion of opportunists or pathogens. Bell et al. (2015) have used a pyrosequencing approach to identify the rhizospheric fungal and bacterial communities associated with willow cultivars in a Zn-polluted soil, and they revealed that during the early growth stages the soil microbiome has the greatest impact on plant function and Zn-extraction (Bell et al., 2015). In this respect, seed-endophytes that early colonize the root and rhizosphere provide an excellent tool to accomplish early rhizosphere colonization, protecting the seedling from exposure to toxic contaminants (Compant et al., 2005; Mastretta et al., 2009; Bragina et al., 2013; Truyens et al., 2013). Recently, Truyens et al. (2015), studied the transgenerational changes in the seed endophytic bacterial community of *Arabidopsis thaliana* exposed to cadmium, and they found that phenotypic characteristics such as cadmium tolerance, the production of phytohormones (auxin), and ACC-deaminase were important selection criteria that were passed from generation to generation (traits is what matters, not who). Also other studies have shown that vertically transmitted seed-endophytic bacteria are an important source for transgenerational plant adaptation to contaminant stress such as cadmium (Mastretta et al., 2009).

Phytoremediation research is also focused on the use of plant-endophytes to improve contaminant biodegradation (Rylott, 2014; Ijaz et al., 2015). The longer contact time between the contaminant and endophytic microorganisms enhances contaminant detoxification thereby reducing the risk of phytotoxicity and otherwise evapotranspiration of volatile organic contaminants (e.g., TCE-dissipation; Barac et al., 2004; Weyens et al., 2009a, 2010a,b). Harnessing the potential of these extraordinary plant-endophyte relationships is not new, though the recent high number of publications reflect the full ongoing extent of this research line (Ijaz et al., 2015). In contrast to rhizospheric strains, endophytes do not have to compete with the large abundance and diversity of (micro)organisms that are present in soil, thereby potentially enhancing their stable establishment and activity. Moreover, introducing species in the endosphere based on the analyses of the core-microbiome reduces the number of uncontrollable environmental factors, to give more reproducible results across environmental settings (Lundberg et al., 2012). In fact, hydrocarbon degradation was more efficient when endophytes were inoculated rather than rhizospheric strains (Andria et al., 2009). The endophytes showed a higher level of root-colonization, gene-expression and maintenance.

Another promising approach to increase the frequency of degradative traits in the rhizosphere microbial community is the transformation of indigenous microorganisms through plasmid introduction. Horizontal transfer of plasmids across species borders makes the stable introduction of cells in high numbers unnecessary (Van Elzas et al., 2003). Moreover the plant-soil interface is a hotspot for horizontal gene transfer because of the various nutrients, and high microbial cell density (Heuer and Smalla, 2012; Wang et al., 2014; Wei et al., 2014), which further encourages cross-species gene transfer (Sentchilo et al., 2013; Wang et al., 2014). Researchers have shown that catabolic plasmids carrying toluene degradation genes were successfully spread from root-endophytes to stem plant-endophytes, that did not previously harbor these plasmids, and this improved the mineralization of toluene and trichloroethylene that would have otherwise been volatilized (Barac et al., 2004; van der Lelie et al., 2005; Weyens et al., 2009a). Plasmid carrying microorganisms have also been added to plant seeds (e.g., yellow lupine), and they have shown to transfer their degradative properties to the surrounding microbiota improving the phytoremediation of nickel and TCE co-contamination (Weyens et al., 2010a). In addition, the use of insects carrying disease-resistant bacteria has also been studied, and these approaches, while not yet introduced for the transfer of catabolic genes, may have applications in phytoremediation research as well (Heuer and Smalla, 2012). In the future, high-throughput metagenome sequencing of extracted plasmid DNA, the ‘mobilome,’ will further advance our insight in the diversity and distribution of plasmid-borne degradation genes (Jorgensen et al., 2014), and metatranscriptomics can be used to identify the response of plants and indigenous microorganisms to plasmid introduction (Zhang et al., 2011).

## Conclusion

Phytoremediation is a promising method for cleaning-up contaminated soils. Experimental evidence underlines the importance of the rhizosphere microbiome in phytoremediation and plant health, and it is clear that the plant is able to control the composition of its microbiome, and by consequence, microbial degradation. The plant host assembles a beneficial microbiome, though trade-offs exist between contaminant degradation and microbes that directly benefit plant growth and health. Therefore, the plant–microbiome may not be optimal and need more targeted human interventions to optimize the plant–microbiome for contaminant degradation. By integrating the current knowledge in a competition-based model, it appears that many new challenges and opportunities have arisen for microbiologists, ecologists, and soil engineers. This is exemplified by the wide diversity of plant-associated microorganisms with potential contaminant detoxifying abilities in the rhizosphere, as well as by difficulties to assess specific host-microbe interactions, and statistical models to elucidate the driving forces. Future approaches in phytoremediation should be focused on the metaorganism rather than single organism-based interventions, and adapt their methods accordingly. Particular emphasis needs to be directed toward selecting plants for their broader interaction with the microbiome and harnessing the nutritional and signaling events between plant and microorganisms. Another strategy is to take advantage of natural gene transfer events which has a strong impact on community composition, contributing to microbial community plasticity. Finally, it could be necessary to disturb the microorganisms in their niche, in order to increase the expression and functionality of catabolic traits. Though, to identify the competitive forces that shape the rhizosphere microbiome and how it affects contaminant biodegradation, both holistic and reductionist approaches should be applied. NGS technologies will become even more than now, an important part of future phytoremediation research, and allows investigating host–microbe interactions at a much higher resolution then before. Perhaps novel insights will lead 1 day to the design of a minimal rhizosphere or plant microbiome for phytoremediation. To conclude, phytoremediation is challenging, but unraveling the mechanisms through which plants control their associated microbiome and vice versa, will open new avenues to enhance phytoremediation efficiency and reliability. In a broader context, understanding how to contain and sustain benefits gained from natural systems is an important endeavor now, and in future, to which we need to contribute, in order to reduce the human impact on the environment and strive toward a more sustainable society.

## Author Contributions

All authors contributed extensively to the work presented in this review. WS and FR provided expertise on the design of the conceptual model for the recruitment of a beneficial microbiome, and helped in editing the manuscript. NW and JV contributed with their profound knowledge concerning the role of plant–microbe interactions during phytoremediation. ST coordinated and wrote this review.

## Conflict of Interest Statement

The authors declare that the research was conducted in the absence of any commercial or financial relationships that could be construed as a potential conflict of interest.
